# Competition between the DNA unwinding and strand pairing activities of the Werner and Bloom syndrome proteins

**DOI:** 10.1186/1471-2199-7-1

**Published:** 2006-01-13

**Authors:** Amrita Machwe, Enerlyn M Lozada, Liren Xiao, David K Orren

**Affiliations:** 1 Graduate Center for Toxicology, University of Kentucky College of Medicine, 800 Rose Street, Lexington, Kentucky, 40536-0305, USA

## Abstract

**Background:**

The premature aging and cancer-prone Werner and Bloom syndromes are caused by defects in the RecQ helicase enzymes WRN and BLM, respectively. Recently, both WRN and BLM (as well as several other RecQ members) have been shown to possess a strand annealing activity in addition to the requisite DNA unwinding activity. Since an annealing function would appear to directly oppose the action of a helicase, we have examined in this study the dynamic equilibrium between unwinding and annealing mediated by either WRN or BLM.

**Results:**

Our investigation into the competition between annealing and unwinding demonstrates that, under standard reaction conditions, WRN- or BLM-mediated annealing can partially or completely mask unwinding as measured in standard helicase assays. Several strategies were employed to suppress the annealing activity so that the actual strength of WRN- or BLM-dependent unwinding could be more accurately assessed. Interestingly, if a DNA oligomer complementary to one strand of the DNA substrate to be unwound is added during the helicase reaction, both WRN and BLM unwinding is enhanced, presumably by preventing protein-mediated re-annealing. This strategy allowed measurement of WRN-catalyzed unwinding of long (80 base pair) duplex regions and fully complementary, blunt-ended duplexes, both of which were otherwise quite refractory to the helicase activity of WRN. Similarly, the addition of trap strand stimulated the ability of BLM to unwind long and blunt-ended duplexes. The stimulatory effect of the human replication protein A (hRPA, the eukaryotic single-stranded DNA binding protein) on both WRN- and BLM-dependent unwinding was also re-examined in light of its possible role in preventing re-annealing. Our results show that hRPA influences the outcome of WRN and BLM helicase assays by both inhibiting re-annealing and directly promoting unwinding, with the larger contribution from the latter mechanism.

**Conclusion:**

These findings indicate that measurements of unwinding by WRN, BLM, and probably other RecQ helicases are complicated by their annealing properties. Thus, WRN- and BLM-dependent unwinding activities are significantly stronger than previously believed. Since this broadens the range of potential physiological substrates for WRN and BLM, our findings have relevance for understanding their functions *in vitro *and *in vivo*.

## Background

Complementary strands of the DNA double helix must be unwound during the course of transcription, replication, recombination, and repair. Therefore, nucleic acid unwinding enzymes (or helicases) have vital functions in almost all DNA metabolic pathways. Helicases of the RecQ family play important roles in maintaining genomic stability, as loss of their individual functions results in chromosomal abnormalities due to increased illegitimate recombination [reviewed in [1,2]]. There are five RecQ helicases in humans, including the WRN, BLM, and RecQ4 proteins that are defective in Werner (WS), Bloom (BS), and Rothmund-Thomson syndromes (RTS), respectively [3-5]. As might be expected from increased generation of chromosomal abnormalities, an elevated frequency of cancer is associated with each of these syndromes; WS is also notable for accelerated development of many other characteristics normally associated with human aging [1,2,6]. Thus, the functions of RecQ helicases in genome maintenance suppress cancer and perhaps other phenotypes associated with aging.

As for most helicases, RecQ family members use energy released during ATP hydrolysis to catalyze unwinding. The directionality of WRN and BLM (as well as all other RecQ members characterized to date) helicase activity is 3' to 5', defined with respect to the DNA strand to which the enzyme is bound and moves along during the unwinding process. With regard to their abilities to bind to and act on various DNA structures, WRN and BLM have been demonstrated to have remarkably similar substrate specificity [reviewed in [2,7]]. Both WRN and BLM have been shown to disrupt Holliday junctions and G-quartets and to unwind duplex regions contained within 3' tailed, forked, and D-loop structures. However, *in vitro *assays suggest that, alone, WRN is a relatively weak helicase, being unable to unwind either fully complementary, blunt-ended duplexes or duplexes longer than about 50–70 bp [8-11]. Although BLM is able to unwind duplexes up to 90 bp, it also cannot unwind fully duplex substrates [11,12]. Notably, addition of human replication protein A (hRPA), the single-stranded DNA binding protein that acts in a number of DNA metabolic pathways, facilitates unwinding of long duplexes (up to 257 bp) by WRN or BLM *in vitro *[8-10]. In contrast to other human RecQ helicases, purified WRN also possesses a 3' to 5' exonuclease activity [13] that may be important for its *in vivo *function. Despite existing knowledge regarding their enzymatic properties, the exact roles of RecQ family members in DNA metabolism remain elusive.

Our laboratory has demonstrated that both WRN and BLM can also facilitate pairing of complementary DNA strands [14]. Recently, these findings with BLM have been confirmed [15] and other RecQ helicases have also been demonstrated to possess similar activities [14,16,17]. Thus, annealing activity is conserved in at least a subset of RecQ helicases. However, it seems likely that annealing mediated by WRN or BLM could act in direct opposition to unwinding, at least during *in vitro *experiments that measure helicase activity on duplex DNA substrates. In this study, the competition between the unwinding and annealing activities of WRN and BLM is examined. Our results indicate that for simple duplex substates, the annealing activity of WRN or BLM can suppress or even completely mask unwinding *in vitro*. To determine the actual strength of WRN- and BLM-dependent unwinding, several strategies were used to preferentially inhibit the annealing reaction. Interestingly, the use of an additional complementary DNA strand (to prevent re-annealing of unwound strands) during helicase reactions revealed that WRN alone could unwind both long and blunt-ended duplexes – structures that had been previously thought to be refractory to WRN helicase activity. Similar results were also achieved with BLM. The addition of hRPA to helicase reactions also stimulated WRN- and BLM-dependent conversion of duplex substrate to single-stranded products, apparently by the combination of inhibiting the re-annealing reaction and directly stimulating the unwinding process. Our findings not only delineate specific strategies to improve the efficacy of assays to measure unwinding by RecQ helicases but also indicate that, when the contributions of protein-dependent annealing are taken into consideration, the helicase activities of both WRN and BLM are significantly stronger than often revealed by direct measurements of unwinding.

## Results and discussion

### Strand pairing activities of WRN and BLM

The RecQ family of helicases has been postulated to be involved in DNA metabolic pathways that require recombinational functions such as pairing of complementary strands and branch migration during heteroduplex formation and resolution. Recent investigations have demonstrated that, in addition to their DNA unwinding activities, a number of RecQ helicases possess an inherent ability to facilitate pairing of complementary DNA strands [14-17]. Our laboratory has demonstrated annealing activity associated with the human WRN and BLM proteins that are deficient in the cancer-prone diseases WS and BS, respectively [14]. In our standard strand pairing assay, the enzyme-mediated conversion of two complementary single-stranded oligomers (one of which is radiolabeled) to a duplex molecule is measured by non-denaturing gel electrophoresis. Since wild type WRN possesses 3' to 5' exonuclease activity that could potentially digest DNA reactants or products, an exonuclease-deficient mutant protein, WRN-E84A, is used here in strand pairing as well as helicase reactions. This WRN-E84A protein will subsequently be referred to as WRN throughout the remainder of this text. It should be noted that in our strand pairing and unwinding assays, a substantial excess of either WRN or BLM is necessary to observe activity. This requirement for excess enzyme might be caused by the presence of significant proportions of inactive molecules in our protein preparations, an oligomeric "active" state for WRN and BLM that necessitates specific minimum protein concentrations, and/or a relatively low activity of WRN and BLM on the single-stranded and duplex structures used for these particular assays. Although we cannot exclude any of these possibilities, it is clear from earlier studies [8-12] and our unpublished results that WRN and BLM act much more efficiently as helicases on very short duplexes than on the longer duplex substrates used in this study. Moreover, both WRN and BLM have much higher affinity for complex DNA structures (forks, D-loops, Holliday junctions, and G quartets) than for either simple single-stranded or duplex substrates [reviewed in [2,7]]. Thus, at least part of the requirement for excess enzyme in these assays is likely due to low affinity for and reduced efficiency on single-stranded and long duplex substrates in comparison to putative physiological DNA substrates – i.e., three- or four-stranded replication or recombination intermediates. It is also probable that, *in vivo*, protein modifications and interactions with other factors enhance the specificity and function of WRN and BLM on their physiological substrates.

**Figure 1:**
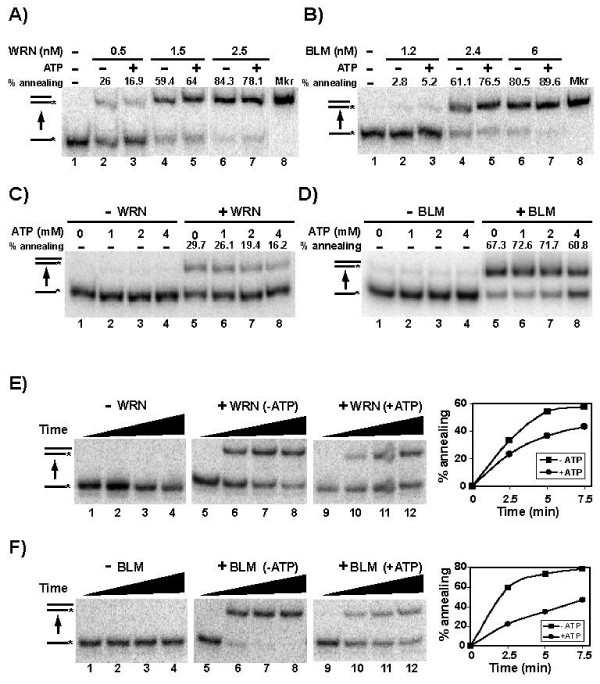
**ATP and DNA substrate structure influence the annealing activities of WRN-E84A and BLM. A) **Labeled C80 (0.025 nM) was incubated for 15 min at 37°C with WRN-E84A (0.5–2.5 nM) and unlabeled G80 (0.025 nM) in the presence or absence of ATP (1 mM) as indicated. These reactions were analyzed as specified in Methods for the strand pairing assay that measures conversion of single-stranded reactants to duplex products (indicated here and subsequently by an upward arrow between DNA structures at left; asterisks show the positions of radiolabels). **B) **Similar experiments were performed as in *A*, except using BLM (1.2–6 nM). **C) **Reactions were performed and analyzed as in *A*, without or with WRN-E84A (0.8 nM) and varying amounts of ATP (0–4 mM) as indicated. **D) **Experiments were performed as in *C*, except with BLM (2.4 nM). For panels *A-D*, the percentage of protein-dependent annealing calculated for each enzyme-containing reaction is given above the relevant lanes. **E) **As in *A*, annealing experiments were performed for the indicated times with WRN-E84A (9 nM), except using unlabeled G95 (0.025 nM) as the complement for labeled C80. Quantitation and graphical representation of the WRN- and BLM-dependent formation of duplex product (including subtraction of protein-independent annealing) in the absence (■) and presence (●) of ATP is depicted at right. **F) **Similar experiments with BLM (3 nM) were performed and analyzed as described in *E*.

The annealing activity for WRN and BLM under our standard conditions on fully complementary 80 nt oligomers in the presence or absence of ATP is demonstrated in Fig. 1A and 1B. Both WRN and BLM, in a protein concentration-dependent manner, mediate efficient conversion of complementary single-stranded oligomers to a blunt-ended duplex. When 1 mM ATP is included and thus unwinding activity is permitted, the pairing of these fully complementary oligomers by WRN and BLM remains surprisingly robust and even comparable to the amount of pairing in the absence of ATP (Fig. 1A and 1B). Since it was possible that limiting amounts of ATP or different Mg^+2^/ATP ratios might influence annealing and unwinding activities, similar reactions were performed with both WRN and BLM again using our standard MgCl_2 _concentration (4 mM) but with the concentration of ATP varying from 0 to 4 mM. The results of these experiments indicate that increasing the amount of ATP in the reaction (or decreasing the Mg^+2^/ATP ratio) slightly decreases the amount of WRN-mediated annealing observed (Fig. 1C). The highest concentration of ATP (4 mM) also had a small inhibitory effect on BLM-mediated annealing of fully complementary oligomers (Fig. 1D). It is unclear whether this effect of increasing ATP concentration is due to inhibition of annealing by the decreased free Mg^+2 ^concentration or to a modest enhancement of unwinding that would decrease the observed extent of duplex formation. Nevertheless, our results suggest that, under conditions that should allow DNA unwinding, the extent of pairing of fully complementary strands mediated by WRN or BLM is not markedly reduced by their helicase activities.

Blunt-ended and long duplex substrates have been demonstrated to be quite refractory to unwinding by WRN or BLM alone [8-11]. Thus, for the experiments above, the forward reaction (annealing) might be much more favored than the back reaction (unwinding) due to the specific structure of the annealing product, a blunt-ended 80 bp duplex, resulting in essentially the same rate and extent of duplex product formation regardless of the presence of ATP. Since substrates with 3' single-stranded tails are reasonably good substrates for the WRN and BLM helicase functions [8,12,18], we postulated that modifiying the strand pairing assay by using one oligomer with additional nucleotides at the 3' end might shift this potential equilibrium toward unwinding. Thus, unlabeled 95 nt and labeled 80 nt oligomers were used in strand pairing reactions with either WRN or BLM such that the potential annealing product would be an 80 bp duplex (with the same sequence as above) but with a 15 nt 3' tail. Time courses were performed to determine the rates of the duplex product formation in the presence and absence of ATP. Under the conditions of these reactions, a minimal amount of protein-independent pairing was observed over a 7.5 min span (Fig. 1E and 1F, *lanes 1–4*). In reactions containing WRN, the amounts of duplex product at each time point are significantly lower when ATP is included (Fig. 1E, *compare lanes 5–8 with lanes 9–12, and right*). With BLM, again a reduced rate of duplex formation with ATP present was observed (Fig. 1F, *compare lanes 5–8 with lanes 9–12, and right*). We conclude that, although the net outcome of these annealing reactions results in formation of double-stranded DNA, inclusion of ATP and the specific structure of the annealing product permits unwinding to occur to some degree, thus lowering the rate and extent of duplex formation. These results suggest that, in reactions containing ATP, the annealing and unwinding activities of WRN and BLM compete when both the single-stranded and duplex DNA structures involved are highly compatible with both activities.

### Stimulation of WRN- and BLM-dependent unwinding of long duplexes

Previous biochemical characterizations have indicated that WRN alone cannot unwind duplexes longer than 50–70 bp [8,9], while BLM is slightly more effective in unwinding long duplexes [10,12]. The discovery of strand pairing activity associated with WRN and BLM and its predominance over unwinding in reactions containing long complementary oligomers suggests a straightforward explanation for the relatively weak unwinding activity that has been observed for these proteins. Specifically, as the length of the duplex region increases, the ability of WRN and BLM to unwind is increasingly outcompeted by their pairing activity, resulting in little or no detectable levels of completely unwound product in standard helicase assays. If this were the case, unwinding of long duplexes might be promoted if the annealing activity of WRN or BLM could be inhibited. Several strategies were considered to minimize or prevent WRN- or BLM-mediated strand pairing.

#### Effect of trap strand

**Figure 2:**
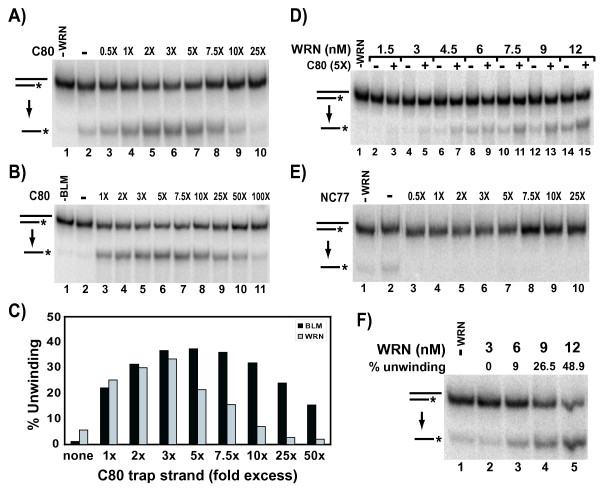
**Addition of trap strand stimulates unwinding of long duplexes by WRN-E84A and BLM. A)** The 80 bp with 15 nt 3’ tail (labeled C80/G95) substrate (0.1 nM) and WRN-E84A (9 nM) without or with the addition of unlabeled C80 oligomer (0.05-2.5 nM, 0.5-25´ with respect to C80/G95 substrate concentration) were incubated at 37^o^C for 7.5 min and DNA products were analyzed as specified in Methods for the helicase assay that measures conversion of duplex reactants to single-stranded products (indicated here and subsequently by a downward arrow between DNA structures at left). **B)** Helicase assays were performed as in A, except using BLM (4 nM) without or with unlabeled C80 (0.1-10 nM, 1-100´). **C)** Quantitation of the stimulation of WRN-E84A (WRN) and BLM unwinding by unlabeled C80 (trap strand) from experiments as those described in A and B.  The WRN and BLM data points are the means of three and four independent experiments, respectively. **D)** Helicase assays were performed as in A, except using variable concentrations of WRN (1.5- 12 nM) and a fixed concentration of unlabeled C80 oligomer (0.5 nM, 5´), respectively. **E)** Helicase assays were performed as in A, except using a non-complementary unlabeled NC77 oligomer (0.05-2.5 nM, 0.5-25´). **F)**  Helicase assays performed without the addition of unlabeled C80, using four-fold lower C80/G95 substrate (25 pM) and WRN-E84A (3-12 nM), with quantitation of WRN-dependent unwinding presented above relevant lanes.

A potential way to inhibit annealing is to trap the completely unwound product/oligomer and prevent it from participating in a WRN- or BLM-mediated annealing reaction. In the standard helicase assay, this might be accomplished by adding a "trap strand" – i.e., an unlabeled oligomer with the identical sequence as the labeled strand of the duplex. Upon unwinding, the unlabeled strand of the duplex becomes paired with and sequestered by this trap strand, preventing its re-annealing with the labeled strand; in this manner, helicase-mediated production of labeled single-stranded product might be maintained. Thus, helicase assays were performed with WRN or BLM and the 80 bp duplex containing a 15 nt 3' tail (labeled C80/G95), with varying amounts (0.5 to 100-fold) of unlabeled oligomer (C80) added just prior to the incubation period. The results of representative experiments for WRN and BLM are shown in Fig. 2A and 2B, respectively, while data derived from multiple experiments is graphed in Fig. 2C. In the absence of trap strand (Fig. 2A, *lane 2*, and 2C), unwinding of this substrate by WRN alone was poor (5.7%). However, addition of the unlabeled C80 trap strand, within a range of concentration from half to 7.5-fold (0.5–7.5×) the molar amount of labeled substrate, markedly stimulated the level of WRN-mediated unwinding (Fig 2A, *lanes 3–9*, and 2C). A trap strand concentration ranging from equimolar to fivefold the amount of substrate optimally stimulated (to a maximum of >30%) WRN unwinding. This stimulatory effect subsided at higher trap strand concentrations with a 25-fold or greater molar excess inhibiting WRN-mediated unwinding. Similar results were observed with BLM (Fig. 2B and 2C). Specifically, there was little or no detectable BLM-mediated unwinding of the 80 bp duplex with the 3' tail without the trap strand, but its addition markedly enhanced unwinding to a maximum of >35% at around a fivefold molar excess of trap strand over substrate. This enhancement of BLM-dependent unwinding was observed over a much wider range of concentration of the trap strand (equimolar to 50-fold excess) than for WRN-dependent unwinding (Fig. 2C). To determine whether this method of stimulation of unwinding was influenced by enzyme concentration, an additional series of unwinding reactions were performed using a fivefold molar excess of trap strand and varying WRN concentrations. The results of these experiments indicate that the stimulation of unwinding of this substrate by addition of trap strand also occurs over a wide range of WRN concentration (Fig. 2D). Importantly, this stimulation of WRN- or BLM-dependent unwinding using a trap strand is not caused by general stimulatory effects of single-stranded DNA on enzyme activity, as addition of the same molar amounts of a completely non-complementary oligomer (NC77) does not stimulate unwinding by either WRN (Fig. 2E) or BLM (data not shown).

#### Effect of DNA substrate concentration

If WRN or BLM pairing ability is masking the detection of their unwinding activity on long substrates, another possible strategy for promoting the unwinding reaction might be to significantly lower the DNA substrate concentration. Since WRN-mediated pairing is a second order reaction dependent on the concentration of each of the complementary strands [14], the rate of pairing would be more dramatically affected than the rate of unwinding (a first order reaction with respect to DNA substrate concentration) by lowering the amount of DNA substrate in the reaction. Accordingly, when helicase assays were performed using one fourth the amount of substrate, significantly higher levels of unwinding (up to 48.9%) of the 3' tailed 80 bp duplex can now be detected without addition of the trap strand (Fig. 2F). These results are the first demonstration that WRN alone can indeed unwind a duplex of at least 80 bp, and conclusively demonstrate that WRN- and BLM-mediated re-annealing tends to directly counteract their abilities to unwind duplex substrates. These findings indicate that, to maximize unwinding, WRN or BLM helicase assays should be performed at the lowest possible DNA substrate concentration and suggest that previous experiments to determine BLM- or WRN-dependent unwinding of long duplexes may have been performed under conditions (for instance, using higher DNA concentrations) in which strand pairing mediated by these same proteins partially or completely masked unwinding.

#### Effect of hRPA

**Figure 3:**
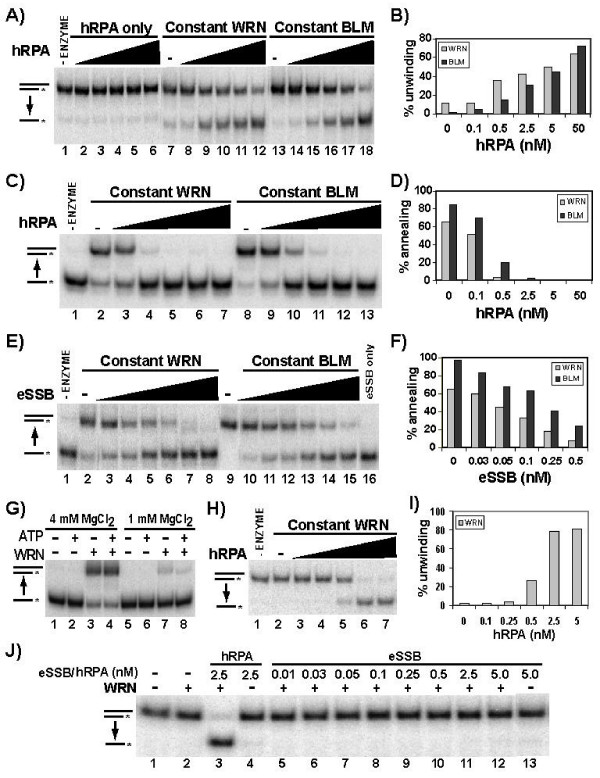
**Effects of hRPA on unwinding and annealing by WRN-E84A and BLM. A) **Helicase reactions were performed for 7.5 min with the 80 bp, 15 nt 3' tail (labeled C80/G95) substrate (0.1 nM) with WRN-E84A (9 nM), BLM (4 nM), and/or hRPA (0.1–50 nM) and analyzed as described in Methods. **B) **Quantitation of data from the experiment depicted in *A*, the effect of hRPA on WRN-E84A and BLM unwinding. **C) **Annealing reactions were performed in the absence of ATP for 7.5 min using labeled C80 (0.1 nM) and unlabeled G95 (0.1 nM) and WRN-E84A (9 nM), BLM (4 nM), and/or hRPA (0.1–50 nM) and DNA products were analyzed as described in Methods. **D) **Quantitation of data from the experiment depicted in *C*, the inhibition of WRN-E84A- (WRN) and BLM-mediated annealing by hRPA. **E) **Annealing assays were performed as in *C*, except using eSSB (0.025–1.25 nM) instead of hRPA. **F) **Quantitation of data from experiments as described in *E*, the inhibition of WRN-E84A (WRN) and BLM-mediated annealing by eSSB. Each data point is the mean of two experiments. **G) **Annealing assays were performed with labeled C80 and unlabeled G95 (0.025 nM each) minus or plus WRN-E84A (4.5 nM) in reaction buffer without or with ATP (1 mM) and either normal (4 mM) or low (1 mM) MgCl_2 _concentration as indicated. **H) **Helicase assays were performed with the 80 bp, 15 nt 3' tail substrate (0.025 nM) in reaction buffer containing 1 mM MgCl_2 _with WRN-E84A (4.5 nM) minus or plus hRPA (0.1–50 nM). **I) **Quantitation of data depicted in *H*, the stimulation of WRN-E84A (WRN) unwinding by hRPA in low (1 mM) MgCl_2_. **J) **Helicase reactions were performed using 1 mM MgCl_2 _as in *H *with or without WRN-E84A (4.5 nM) and the indicated concentrations of hRPA or eSSB.

The unwinding of long duplexes up to 257 bp (constructed by annealing denatured fragments to a large single-stranded circular M13 DNA) by WRN or BLM can be achieved by the addition of hRPA, the heterotrimeric single-stranded DNA binding protein complex involved in many DNA metabolic pathways [8-10]. Since our experiments indicate that the pairing activities of WRN and BLM tend to counter unwinding activity, one possible mechanism of this hRPA stimulation might be to prevent the re-annealing of complementary strands by its single-stranded DNA binding capability. In order to test this hypothesis, experiments were first performed to determine the effect of hRPA on unwinding of our oligomer-based substrates. Alone, hRPA had no detectable unwinding activity and, as above, WRN or BLM alone show very limited unwinding of this substrate (Fig. 3A). However, the addition of hRPA dramatically stimulated the level of WRN and BLM unwinding of our 80 bp substrate with 15 nt 3' tail (Fig. 3A and 3B). This stimulation of WRN- or BLM-mediated unwinding was evident at a hRPA concentration of 0.5 nM (a fivefold molar excess over the DNA substrate concentration, 2.5-fold with respect to the amount of single-stranded DNA that could potentially be produced) and gradually increased with higher hRPA concentrations. These results indicate that hRPA-mediated stimulation of WRN- and BLM-dependent unwinding operates not only with M13-based circular substrates but also with oligomeric substrates.

Next, the effect of hRPA on WRN- or BLM-dependent pairing of complementary oligomers (G95 and C80) was examined in the absence of ATP. In these reactions, WRN or BLM alone facilitated annealing of the oligomers as expected (Fig. 3C, *lanes 2 and 8*, and 3D). At a hRPA concentration of 0.1 nM, both WRN- and BLM-dependent pairing were modestly inhibited, while hRPA concentrations of 0.5 nM and above completely inhibited WRN- and BLM-mediated pairing (Fig. 3C, *lanes 3–7 and 9–12*, and 3D). One heterotrimeric hRPA complex binds to about 30 nt of single-stranded DNA [19]; considering that the mean length of these oligomers is 87.5 nt, each oligomer can bind about 3 hRPA molecules. Thus, with respect to the combined oligomer concentration (0.2 nM), a threefold excess (0.6 nM) of hRPA should be sufficient to completely cover all of the single-stranded DNA in these reactions, an amount that is in good agreement with the hRPA concentrations observed to almost completely inhibit pairing (Fig. 3C and 3D). Thus, the direct binding of single-stranded oligomers by hRPA would appear to be responsible for its inhibition of WRN- and BLM-mediated annealing. It also seems likely that at least some of hRPA's positive effect on WRN- and BLM-dependent unwinding of long oligomers is due to its inhibition of the competing back reaction, annealing. When *Escherichia coli *single-stranded binding protein (eSSB), the prokaryotic equivalent of hRPA, was added to these annealing reactions, a similar pattern of inhibition of WRN- or BLM-mediated pairing was observed (Fig. 3E and 3F). Specifically, either WRN or BLM annealing activity was progressively inhibited by increasing concentrations of eSSB between 0.05–0.5 nM, a slightly lower molar range than observed for inhibition by hRPA.

From the experiments above, it is clear that the molar amount of hRPA that completely inhibited pairing was significantly less than the amount that maximally promoted unwinding, suggesting that the stimulatory effect of hRPA on WRN- or BLM-dependent unwinding was not simply due to inhibition of annealing. To further examine this question, we decided to test whether hRPA still stimulates unwinding under conditions in which the pairing reaction is minimal or non-existent. Our characterization of the strand pairing activities of WRN and BLM indicated that the divalent cation concentration has a marked effect on protein-mediated annealing [14]. Specifically, pairing of 80-mers by WRN was optimal at Mg^+2 ^concentrations of between 4–20 mM and drops precipitously at concentrations lower than 4 mM. Notably, BLM-mediated annealing appears to be significantly less sensitive to low MgCl_2 _concentrations than WRN [14,15]. Our results above (see Fig. 2F) also suggest that pairing is more affected by DNA substrate concentration than unwinding. Although Mg^+2 ^is certainly required for ATPase and helicase activity, we postulated that a lower DNA concentration and a low Mg^+2 ^concentration (1 mM) might permit unwinding while almost completely inhibiting pairing. In experiments to compare WRN annealing activity in the absence and presence of ATP at low (1 mM) and our standard (4 mM) Mg^+2 ^concentrations, clearly WRN-dependent pairing was almost completely inhibited at 1 mM MgCl_2 _whether or not ATP was present (Fig. 3G). Subsequently, WRN unwinding reactions containing the 80 bp substrate with 15 nt 3' tail were performed in 1 mM MgCl_2 _and 1 mM ATP with or without hRPA. The results of these experiments (Fig. 3H and 3I) show no unwinding by WRN alone or in the presence of hRPA at concentrations of 0.25 nM and below. However, WRN-dependent unwinding is observed at a hRPA concentration of 0.5 nM, and nearly complete at hRPA concentrations of 2.5 nM and above. Similar reactions were also performed at 1 mM MgCl_2 _and 1 mM ATP with WRN plus or minus eSSB (Fig. 3J). While a positive control showed that hRPA again stimulated WRN-dependent conversion of duplex to single-stranded DNA (Fig. 3J, *lane 3*), WRN-mediated unwinding was not detected upon addition of eSSB over a wide range of concentration (Fig. 3J, *lanes 5–13*), including amounts that were capable of completely inhibiting annealing (see Fig. 3E and 3F). Since re-annealing (the back reaction) is minimal under these conditions, the stimulation observed with hRPA is due to a direct and specific effect on WRN-catalyzed unwinding that cannot be reproduced using the heterologous single-stranded binding protein eSSB. If these results are compared with those above (Fig. 3A–D) using 4 mM MgCl_2_, it appears that the enhanced formation of single-stranded products at low hRPA concentrations (< 2.5 nM) is due to inhibition of re-annealing while higher hRPA concentrations directly stimulate the unwinding reaction. It also appears that some of the stimulatory effect of hRPA between 2.5–50 nM is somewhat tempered by WRN-mediated pairing at the higher Mg^+2 ^concentration. Nevertheless, our results indicate that, at least under our standard Mg^+2 ^conditions, hRPA affects the outcome of helicase assays by both enhancing unwinding and inhibiting re-annealing.

### WRN and BLM can unwind blunt-ended duplexes

**Figure 4:**
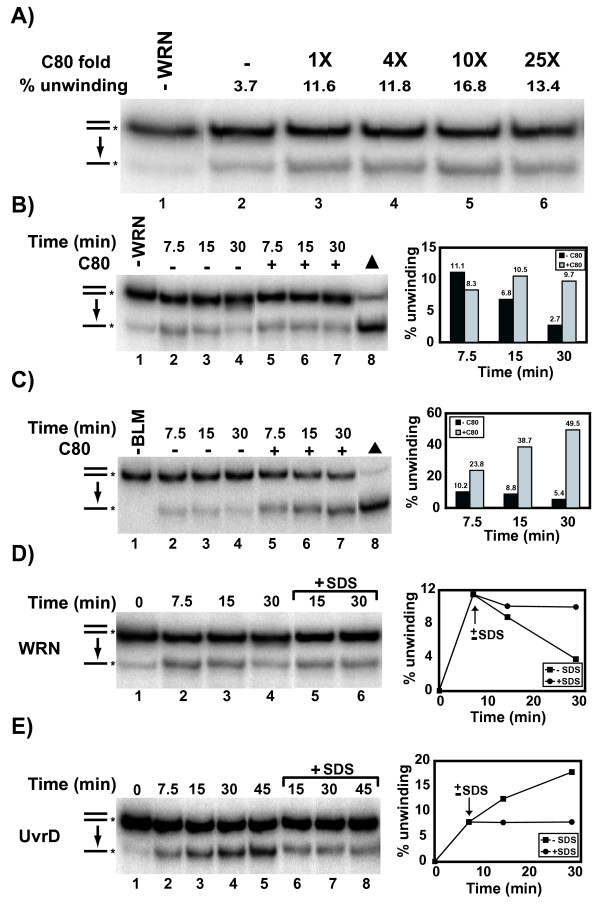
**Unwinding of blunt-ended duplex substrates by WRN-E84A and BLM. A) **Helicase reactions containing 2 mM ATP, fully duplex (labeled C80/G0) substrate (0.5 nM) and WRN-E84A (96 nM) were incubated at 37°C, with or without addition of unlabeled C80 (0.5–12.5 nM) at 3 min into the incubation. Reactions were terminated after 15 min total and DNA products analyzed as described in Methods. Quantitation of the amount of WRN-dependent unwinding is presented above lanes 2–6. **B) **Parallel helicase reactions were performed as in *A *using WRN-E84A (96 nM) with or without unlabeled C80 (5 nM) added 3 min into the reaction. For each reaction, 0, 7.5, 15, and 30 min time points were taken and DNA products analyzed as above. Quantitation of the level of unwinding in reactions with or without unlabeled C80 appears at right. **C) **Parallel helicase reactions containing labeled C80/G80 (0.05 nM), BLM (12 nM) with or without unlabeled C80 (0.2 nM) were performed, analyzed, and depicted graphically as in *B*. **D) **Parallel helicase reactions containing fully duplex substrate (0.5 nM) and WRN-E84A (77 nM) were incubated at 37°C with or without addition of SDS (1%) at 7.5 min into the incubation. Time points taken at 0, 7.5, 15, and 30 min were analyzed as above. Quantitation of the percentage of unwound product over time appears at right. **E) **Time points (0, 7.5, 15, and 30 min) from parallel helicase reactions are analyzed and depicted as in *D*, except using UvrD (14 nM) instead of WRN-E84A.

The experiments above suggest that the outcomes of *in vitro *assays to measure unwinding, particularly of long duplexes, by WRN or BLM are complicated or even compromised by their ability to readily re-anneal the unwound strands. The strategy of inhibiting the pairing reaction allowed visualization of WRN-catalyzed unwinding of long duplexes that could not be observed otherwise; BLM-catalyzed unwinding was also similarly enhanced. Although earlier reports suggested that fully complementary, blunt-ended duplexes are not substrates for WRN- or BLM-mediated unwinding [8,11], we considered the possibility that, in these instances, any unwinding might have been completely masked by the counteracting pairing reaction. To determine whether this was the case, we performed unwinding assays using a fully duplex 80 bp substrate and adopted the strategy of adding a trap strand to the reaction. Preliminary assays in which the trap strand was added at the start of the incubation did not permit WRN-dependent unwinding (data not shown). Since it was possible that, in these reactions, the trap strand outcompeted the duplex substrate for initial WRN binding, another series of experiments were performed in which WRN and the duplex substrate were pre-incubated for 5 min at 4°C and the reactions were incubated at 37°C for 3 min before addition of the trap strand. Theoretically, this experimental design should favor the binding of the duplex substrate and initiation of its unwinding by WRN and the later addition of trap strand may still be able to inhibit subsequent WRN-mediated re-annealing. When unwinding reactions were performed in this manner, the addition of trap strand modestly stimulated the amount of WRN-mediated unwinding of the fully duplex substrate over a 15 min interval (Fig. 4A). This stimulation was observed at an equimolar concentration up to a tenfold excess of trap strand with respect to the DNA substrate concentration. As above, this effect was specific for trap strand that is fully complementary, as addition of a non-complementary oligomer did not stimulate WRN unwinding (data not shown).

These results indicate that WRN has the ability to unwind a fully duplex substrate. Since the trap strand unmasked this activity only after the unwinding reaction was initiated, kinetic experiments were performed to examine the competition between the unwinding and annealing reactions. In these experiments (Fig. 4B), WRN-mediated unwinding was measured over 30 min, without or with the addition of a tenfold molar excess of trap strand at 3 min into the reaction. In the absence of trap strand, the amount of substrate unwound by WRN is highest at the first time point taken (7.5 min) and decreases thereafter, returning essentially to background by 30 min. When trap strand is present, the amount of WRN-mediated unwinding remained relatively constant over the 30 min period. In similar experiments performed with BLM (Fig. 4C), the amount of unwinding of blunt-ended duplex by BLM decreases with time when the trap strand is not present, as observed with WRN. However, the addition of a four-fold molar excess of trap strand at 3 min into the reaction resulted not only in stabilizing but also gradually increasing the amount of BLM-dependent unwinding throughout the time course.

To confirm that this time-dependent re-annealing was also mediated by WRN, parallel unwinding reactions were performed in the absence of a trap strand, with the protein denaturant SDS added to one reaction at 7.5 min into the incubation (Fig. 4D). SDS essentially kept the amount of unwinding at the level measured at the time of its addition, while the amount of unwinding in reactions without SDS again decreased over time. These results indicate that, although WRN is mediating some unwinding of this fully duplex substrate early in the time course, any unwinding is eventually countered by WRN-mediated re-annealing of the unwound strands. When an identical experiment was performed with the *Escherichia coli *UvrD protein, a 3' to 5' helicase from outside the RecQ family, again SDS completely abolished enzymatic activity and the amount of unwinding remained constant after its addition (Fig. 4E). However, in contrast to WRN, the amount of unwinding catalyzed by UvrD in reactions without addition of SDS increased steadily over a 45 min interval, suggesting that the dynamic unwinding and re-annealing equilibrium is specific for WRN, BLM, and perhaps other RecQ helicases but not common to all unwinding enzymes. Taken together, these results demonstrate that, over time, WRN- and BLM-mediated unwinding is reversed by re-annealing unless one of the unwound strands (here the unlabeled strand of the substrate) is sequestered by the addition of a complementary trap strand. Our findings also indicate that, if measures are taken to minimize the annealing reaction, WRN- and BLM-mediated unwinding is enhanced to a degree previously not observed.

## Conclusion

Several RecQ helicases, including WRN and BLM, have recently been demonstrated to have annealing properties in addition to their requisite unwinding activities [14-17]. In theory and practice, unwinding and annealing activities within the same protein would tend to work against one another. Here, we have examined this competition between the unwinding and annealing activities of WRN and BLM *in vitro*. Under reaction conditions that permit both activities (utilization of appropriate DNA structures plus the inclusion of ATP), annealing counters unwinding and vice versa. The balance between these activities depends on 1) DNA concentration, as lower concentrations favor the first order unwinding reaction as opposed to the second order annealing reaction, 2) the length of duplex or regions of complementarity, with annealing being favored as this length increases, 3) the structures of the DNA substrates to be unwound or paired, 4) divalent cation concentration or Mg^+2^/ATP ratios, with lower Mg^+2 ^concentrations (and thus lower Mg^+2^/ATP ratios) reducing the relative contributions of annealing, and 5) enzyme concentration. With regard to Mg^+2 ^concentration (and/or the Mg^+2^/ATP ratio), it appears that the effect of these variables on the balance between annealing and unwinding is influenced by substrate structure and length. In comparison to our results demonstrating that WRN and BLM annealing of fully complementary 80-mers is markedly diminished at low Mg^+2 ^concentrations [[14] and this study], Cheok et al. have observed a much smaller effect of Mg^+2 ^concentration on BLM-mediated annealing of shorter, partially complementary oligomers [15]. Despite these differences, both studies agree in that BLM-mediated annealing appears to be significantly more resistant to changes in Mg^+2 ^concentration than WRN-mediated annealing. Although increasing enzyme (WRN) concentrations in reactions containing 80 bp substrates increases levels of unwinding (see Fig. 2F), we expect that the effect of enzyme concentration on the balance between unwinding and annealing will be complex and vary based on the structure of DNA substrates including duplex length. Indeed, Cheok et al. [15] have very recently shown that increasing BLM concentration actually reduces the net unwinding of a 31 bp forked substrate due to an increase in BLM-dependent annealing. Depending on the DNA substrates and reaction conditions, the ability to measure WRN or BLM unwinding can be diminished or completely masked by their propensity to readily re-anneal the unwound strands. Thus, unwinding assays performed with WRN or BLM should be designed and analyzed with consideration of the effect of protein-mediated re-annealing.

With the knowledge that annealing counteracts unwinding, strategies were attempted to minimize or eliminate the protein-mediated annealing reaction and thus obtain greater insight into the strength of WRN- or BLM-dependent unwinding. In summary, our strategies to reduce protein-mediated re-annealing demonstrate that WRN and BLM are significantly more robust helicases than revealed by previous measurements. One strategy employed to diminish the contribution of annealing, the utilization of trap strand, was surprisingly effective considering the likelihood that addition of a single-stranded oligomer would compete with DNA substrate for enzyme binding. Although this potential competition of trap strand with substrate may indeed affect the unwinding reaction, the trap strand appears to have a more profound inhibitory effect on the annealing reaction, resulting in a net stimulatory effect on the outcome of helicase assays. Significant unwinding of blunt-ended duplexes that had not been observed previously using WRN or BLM alone was revealed when trap strand was added following initiation of the enzymatic reaction. Our results also show that a significant excess of complementary single-stranded 80 nt oligomer added prior to the start of a helicase reaction dramatically stimulates unwinding of an 80 bp duplex with a 3' tail. The latter results would suggest that the affinity of WRN or BLM for the junction between single- and double-stranded DNA in the substrate is significantly greater than their affinity for single-stranded DNA alone. This is in general agreement with another report that suggests preferential targeting of WRN to duplex junctions (i.e., forked structures) over simple single-stranded regions within a single DNA substrate [18]. Notably, at very high concentrations of trap strand this stimulation subsides, probably due to a decreasing ability of WRN or BLM to find and bind to the duplex substrate amid a vast excess of single-stranded DNA. Another possible explanation for this stimulation by trap strand is that WRN and BLM have the capability to scan a large amount of both single- and double-stranded DNA very rapidly, and thus can still access and unwind duplexes with the products subsequently sequestered by the trap strand. Yet a third and most intriguing possibility exists – i.e., that the trap strand can actually participate in and promote a WRN- or BLM-mediated strand invasion event that results in strand exchange and the appearance of unwinding in our helicase assays. Notably, we have previously observed WRN- and BLM-mediated strand exchange [14]. However, in contrast to the experiments conducted in this study with duplex substrates without single-stranded regions complementary to the trap strand, the original substrate in our earlier study contained a single-stranded region that could have served as the initiation target for annealing and the subsequent strand exchange process. Further experimentation is necessary to determine whether WRN or BLM can initiate a strand invasion event within a fully duplex region in a manner similar to bacterial RecA or eukaryotic RAD51 protein.

Addition of hRPA stimulates WRN- and BLM-mediated unwinding of long duplex regions contained within large M13 single-stranded circles [8-10]. When this strategy was employed using our 80 bp oligomer-based substrate containing a 3' tail, hRPA again markedly stimulated both WRN- and BLM-dependent unwinding. Since our substrate contained only 15 nt of single-stranded DNA, this finding eliminates the possibility that hRPA stimulation of unwinding in previous studies might have been due to its binding to the large single-stranded regions present on M13-based substrates and thus reducing potential non-specific and non-productive binding of WRN or BLM. Since WRN and BLM have strong annealing properties, another possibility for this hRPA stimulation could be inhibition of re-annealing following initial unwinding. A direct test of hRPA's effect on annealing alone indicated that, indeed, hRPA dramatically inhibited WRN- and BLM-mediated annealing. However, the concentrations of hRPA that optimally stimulated unwinding were significantly higher than the concentrations that inhibited annealing, suggesting that two different mechanisms might contribute to the increased amounts of single-stranded product in helicase reactions containing hRPA. Furthermore, hRPA still dramatically stimulates WRN unwinding at low MgCl_2 _concentrations that dramatically suppress WRN annealing activity, suggesting that hRPA directly promotes the unwinding reaction. Thus, during helicase assays performed at standard Mg^+2 ^concentrations, hRPA increases the production of unwound DNA strands by both inhibiting re-annealing and stimulating unwinding. However, hRPA's positive influence on the unwinding reaction appears to account for most of its stimulatory effect. This is further supported by our results with eSSB, which also dramatically inhibits WRN- and BLM-mediated annealing but does not stimulate unwinding of our 80 bp, 15 nt 3' tail substrate. These results are in general agreement with the weak enhancement of WRN- and BLM-dependent unwinding of M13-based duplexes by heterologous single-stranded binding proteins [8-10]. The mechanism of hRPA's direct and specific stimulatory effect would appear to be consistent with its delivery to single-stranded DNA at the site of WRN- or BLM-mediated unwinding through direct protein-protein interactions observed previously [9,10].

The *in vitro *competition between the unwinding and annealing activities of WRN and BLM demonstrated here and likely applicable to other RecQ helicases with annealing activity (including human RecQ1 and human RecQ5β) would seem to be counterproductive from a physiological point of view. Annealing activity by human RecQ helicases might also interfere with the stabilization of single-stranded DNA by hRPA (or other proteins) that occurs as part of normal DNA metabolism. However, as observed in this study, hRPA (and heterologous eSSB) outcompetes WRN and BLM for single-stranded DNA and thus prevents WRN- or BLM-mediated annealing. For this and other reasons, we do not expect that either WRN or BLM acts to simply pair complementary strands that might arise as a result of an unrelated DNA metabolic event. We suggest two scenarios that might explain the presence and need for both activities in a single enzyme. In the first scenario, WRN- or BLM-mediated re-annealing following unwinding limits the extent of the unwound region in the context of long duplexes. This could be important for minimizing attack of the unwound regions of single-stranded DNA by nucleases or other proteins. However, this runs counter to the observation, shown here and in earlier reports [8-10], that hRPA promotes WRN- and BLM-dependent unwinding and stabilizes the resulting single-stranded DNA. We favor another scenario, one in which WRN- or BLM-catalyzed unwinding and annealing act concertedly on complex three- and four-stranded DNA structures to perform strand exchange and/or branch migration. In fact, strand exchange resulting from the coordination of WRN- or BLM-mediated annealing and unwinding has been previously demonstrated by our laboratory [14]. Such coordination of annealing and unwinding activities could be potentially involved in regression of replication forks or formation or resolution of heteroduplexes during recombination reactions. Importantly, resolution of replication blockage utilizing fork regression combined with either repair or recombinational pathways appears to be a critical metabolic process for both permitting completion of replication and maintaining genome stability [reviewed in [20-24]]. The potential coordination between the unwinding and annealing activities of WRN or BLM (or other RecQ members) to facilitate replication fork regression, strand exchange and/or branch migration would seem to be most consistent with the illegitimate recombination phenotypes of RecQ-deficient cells and the proposed roles for RecQ helicases in recombinational repair.

## Methods

### Proteins

The WRN-E84A protein that lacks 3' to 5' exonuclease activity but retains ATPase and helicase activity was overexpressed and purified as described previously for wild-type WRN [25], except for the use of 0.1% Nonidet-P40 (NP40) in all liquid chromatography buffers. The BLM protein, a kind gift from J. Groden (University of Cincinnati), was overexpressed and purified essentially as described by Karow et al. [12]. Purified UvrD and hRPA were kind gifts from S. Matson (University of North Carolina at Chapel Hill) and G.-M. Li (University of Kentucky), respectively. eSSB was purchased from US Biochemicals.

### DNA substrates

Oligonucleotides C80, G80, and G95 were purchased from Integrated DNA Technologies (Coralville, IA) and their sequences, in 5' to 3' orientation, are as follows: *C80 *= GCTGATCAACCCTACATGTGTAGGTAACCCTAACCCTAACCCTAAGGACAACCCTAGTGAAGCTTGTAACCCTAGGAGCT, *G80 *= AGCTCCTAGGGTTACAAGCTTCACTAGGGTTGTCCTTAGGGTTAGGGTTAGGGTTACCTACACATGTAGGGTTGATCAGC, *G95 *= AGCTCCTAGGGTTACAAGCTTCACTAGGGTTGTCCTTAGGGTTAGGGTTAGGGTTACCTACACATGTAGGGTTGATCAGCTACGTCATGCTCAGA, and *NC77 *= AGCTGAGCATGTCCAGACATGTCCGTGAGTGTGAGTGTGAGTGTGAGTGTGAGTGTGAGTGTGAGTGTGAGTGTGAG. The C80 oligomer was radiolabeled at its 5' end with ^32^P-γ-ATP (3000 Ci/mmol) and polynucleotide kinase, 3' phosphatase-free (Roche Biochemicals) and unincorporated nucleotides were removed using standard procedures. For construction of the blunt-ended or 15 nt 3' tail 80 bp substrates, labeled C80 was annealed to a twofold excess of unlabeled G80 or G95, respectively. After separation by non-denaturing polyacrylamide (12%) gel electrophoresis (PAGE), labeled oligomers and annealed duplex substrates were then purified using a gel extraction kit (Qiagen). The concentration of labeled, purified C80 was determined from the amount of unlabeled complementary G80 required to completely convert this oligomer to duplex over a 24 h period; concentrations of duplex substrates were then calculated using the specific activity of the isotope.

### Helicase assay

To measure enzyme-catalyzed unwinding, the fully duplex (labeled C80/G80) substrate (0.05–0.5 nM) or the 80 bp duplex with 15 nt 3' tail (labeled C80/G95) substrate (0.025–0.1 nM) was incubated without or with WRN-E84A (1.5–96 nM), BLM (4–12 nM), or UvrD (14 nM) in reaction buffer [40 mM Tris-HCl (pH 8.0), 4 mM MgCl_2_, 0.1 mg/ml bovine serum albumin, 0.1% NP40, and 5 mM dithiothreitol] supplemented with ATP (1 mM, unless otherwise stipulated) at 37°C for the specified times. For certain experiments, a lower MgCl_2 _concentration (1 mM) was used in the reaction buffer. Where indicated, reactions also contained unlabeled single-stranded C80 (0.05–12.5 nM) or NC77 oligomer (0.5–2.5 nM), hRPA (0.1–50 nM), or eSSB (0.01–5 nM). When present, the unlabeled oligomers were added as specified in the text and/or figure legends, either prior to incubation at 37°C or 3 min following initiation of the reaction at 37°C. For specific experiments, SDS was added to a final concentration of 1% at 7.5 min into the incubation period. Helicase reactions were terminated by adding one-sixth volume of helicase dyes (30% glycerol, 50 mM EDTA, 0.9% SDS, 0.25% bromphenol blue and 0.25% xylene cyanol) including excess unlabeled C80 to prevent potential protein-independent annealing following the enzymatic reaction interval. DNA products were separated by non-denaturing (8%) PAGE and, after gel drying, were visualized and quantitated using a Storm 860 phosphorimager with ImageQuant software (Molecular Dynamics). Percent unwinding is calculated as [the amount of single-stranded species divided by the total amount of labeled DNA in each lane (duplex plus single-stranded species)] × 100, including subtraction of any single-stranded component in the original substrate preparation.

### Strand pairing assay

To measure protein-mediated annealing, 5'-radiolabeled C80 oligomer (0.025–0.1 nM) without or with WRN-E84A (0.5–9 nM) or BLM (1.2–6 nM) and either G95 (0.025–0.1 nM) or G80 (0.025–0.1 nM) in reaction buffer is incubated at 37°C for the times specified in the figure legends. Where indicated, the reactions also contained hRPA (0.1–50 nM), eSSB (0.025–1.25 nM), a lower MgCl_2 _concentration (1 mM) and/or variable amounts of ATP (1, 2, or 4 mM). Pairing reactions were terminated and DNA products were separated and analyzed as described above for the helicase assay. Protein-dependent strand pairing (% annealing) is calculated as [the amount of duplex produced divided by the total amount of labeled DNA in each lane (duplex plus single-stranded species)] × 100, including subtraction of a very small percentage of protein-independent annealing in reactions containing the complementary oligomers without either WRN or BLM.

## Abbreviations

BS: Bloom syndrome, eSSB: *Escherichia coli *single-stranded binding protein, hRPA: human replication protein A, NP-40: Nonidet P-40, PAGE: polyacrylamide gel electrophoresis, SDS: sodium dodecyl sulfate, WS: Werner syndrome

## Authors' contributions

AM participated in the design of the experiments and performed specific biochemical assays, prepared materials for publication, and helped draft the manuscript. EML performed biochemical assays and prepared materials for publication. XL carried out biochemical assays and prepared DNA substrates and purified proteins. DKO conceived of the study, oversaw the design of all experiments, and participated in drafting the manuscript. Each author has read and approved the final version of the manuscript.
